# Supporting data of spatiotemporal proliferation of human stromal cells adjusts to nutrient availability and leads to stanniocalcin-1 expression *in vitro* and *in vivo*

**DOI:** 10.1016/j.dib.2015.08.012

**Published:** 2015-08-28

**Authors:** Gustavo A. Higuera, Hugo Fernandes, Tim W.G.M. Spitters, Jeroen van de Peppel, Nils Aufferman, Roman Truckenmueller, Maryana Escalante, Reinout Stoop, Johannes P van Leeuwen, Jan de Boer, Vinod Subramaniam, Marcel Karperien, Clemens van Blitterswijk, Anton van Boxtel, Lorenzo Moroni

**Affiliations:** a Department of Tissue Regeneration, MIRA - Institute for Biomedical Technology, University of Twente, Drienerlolaan 5, 7522 NB, Enschede, The Netherlands; b Erasmus Medical Center, Internal Medicine, Wytemaweg 80, 3015 CN Rotterdam, The Netherlands; c Biophysical Engineering Group, Mesa^+^ Institute for Nanotechnology, University of Twente, Drienerlolaan 5, 7522 NB Enschede, The Netherlands; d TNO, Metabolic Health Research, Zernikedreef 9, 2333 CK Leiden, The Netherlands; e Systems and Control Group, Wageningen University, PO Box 17, 6700 AA Wageningen, The Netherlands; f Department of Complex Tissue Regeneration, MERLN Institute for Technology Inspired Regenerative Medicine, Universiteitsingel 40, 6229ER Maastricht, The Netherlands; g Department of cell biology inspired tissue engineering, MERLN Institute for Technology Inspired Regenerative Medicine, Universiteitsingel 40, 6229ER Maastricht, The Netherlands

## Abstract

This data article contains seven figures and two tables supporting the research article entitled: spatiotemporal proliferation of human stromal cells adjusts to nutrient availability and leads to stanniocalcin-1 expression *in vitro* and *in vivo*[1]. The data explain the culture of stromal cells *in vitro* in three culture systems: discs, scaffolds and scaffolds in a perfusion bioreactor system. Also, quantification of extracellular matrix components (ECM) *in vitro* and staining of ECM components *in vivo* can be found here. Finally the quantification of blood vessels dimensions from CD31 signals and representative histograms of stanniocalcin-1 fluorescent signals in negative controls and experimental conditions *in vivo* are presented.

**Specifications Table**

**Table:** 

Subject area	Physics, Biology
More specific subject area	Stromal cell culture and therapy
Type of data	Table, image (microscopy), text file, graph, figure
How data was acquired	Confocal microscope, SEM, µCT, perfusion bioreactor, oxygen sensors
Data format	Analyzed
Experimental factors	Human stromal cells were cultured in 2D, 3D, under shear stress, concentrated in implantable wells and implanted in mice.
Experimental features	See experimental details for each figure
Data source location	The Netherlands
Data accessibility	Within this article

**Value of the data**

• Human stromal cell proliferation under various conditions.
• Multidisciplinary approach to understand and exploit the therapeutic potential of cells.
• Results of implantation in mice of human cells in a novel well system.

## 1 Data, experimental design, materials and methods

### 1.1 Scaffold characterization

**Fig. 1:**
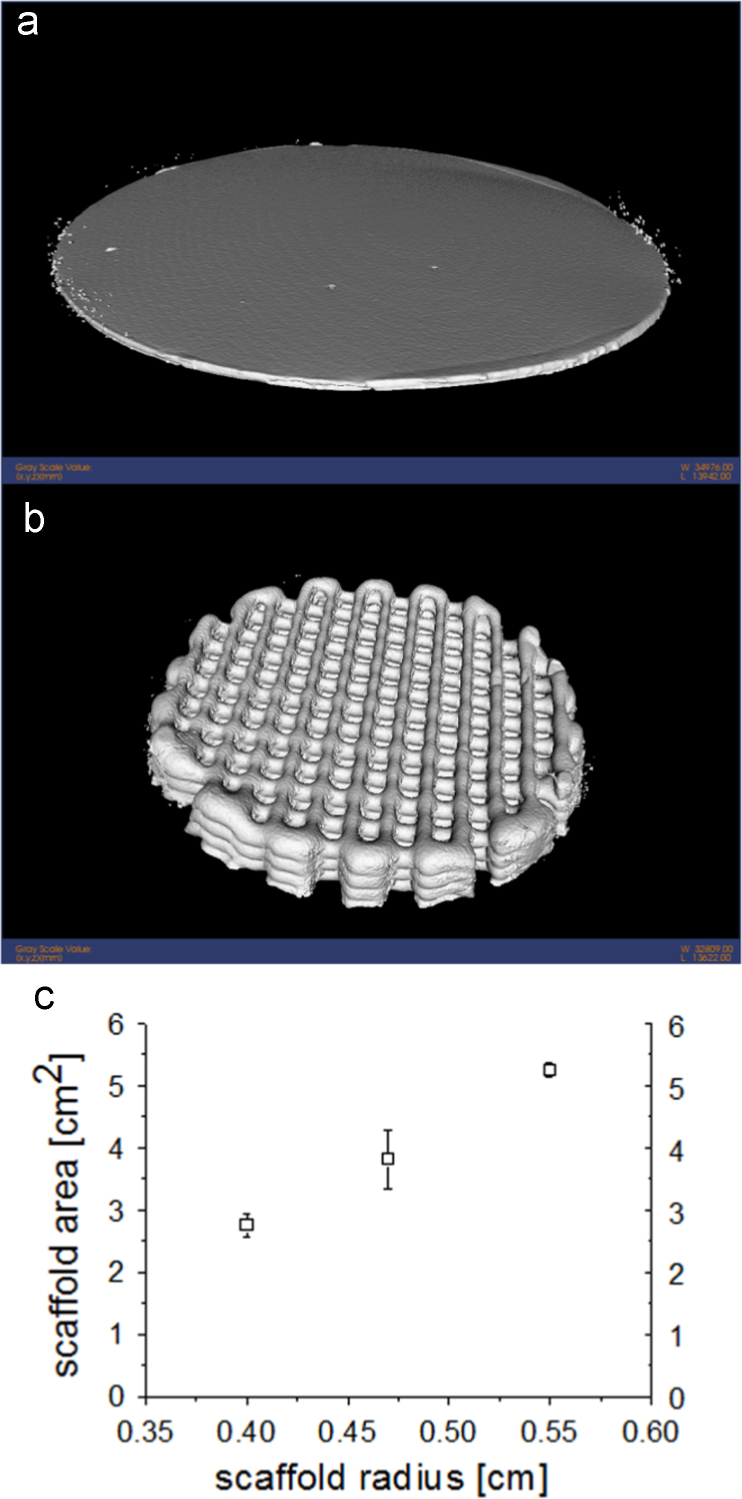
2D and 3D culture systems and surface area. Representative image acquired through µCT of the PEOT/PBT films (A) and scaffolds (B). The radius of scaffolds is plotted against the surface area measurements (C), which allowed the production of 2D and 3D systems of PEOT/PBT with comparable surface areas.

Microcomputed tomography (μCT, eXplore Locus SP μCT scanner, GE, Brussels, Belgium) at 14 μm resolution were used to characterize 2D and 3D scaffolds. Volume, porosity, and surface area of 2D and 3D scaffolds were determined with Microview software (Open source) as performed before [2]. Briefly, the threshold was adjusted to differentiate on the grayscale image between polymer voxels and pore voxels (One voxel was a 23×23×23 μm volume-element). The fraction of pore voxels within a scaffold determined its porosity. The pore size was determined by filling pore voxels with overlapping spheres [3]. The average size of a sphere occupying the pore voxel determined the average pore size. The boundaries between pore and polymer voxels determine the specific surface area.(Fig. 1 and Tables 1 and 2)

### 1.2 Perfusion bioreactor culture

A direct perfusion flow bioreactor configuration was used. The bioreactor was comprised of inner and outer housings made of polycarbonate (Applikon Biotechnology BV, Schiedam, The Netherlands), where PEOT/PBT cylindrical scaffolds (8 mm in diameter by 3 mm in height) were kept press-fit in the inner housing during cultivation. The bioreactor was connected to 3.2 mm PharMed tubing (Cole-palmer, The Netherlands), which was used throughout the system in a loop composed of: a supply vessel, 0.89 mm microbore tubing (Cole-palmer) used only on a pumphead (Masterflex, the Netherlands), fittings to connect 0.89–3.2 mm tubing, an oxygenator (explained below), the bioreactor, in-line oxygen and pH microsensors (Presens GmbH, Germany) and back to the supply vessel. One run was defined by four of these systems run in parallel for 8 days with medium refreshments twice a week, where each system contained an 8×3 mm cylindrical scaffold dynamically seeded with 1.5 million cells and connected independently to their own oxygenator, tubing and αMEM proliferation medium supply.

To achieve flows in the range of 0.1–1 ml/min, a pump head (Masterflex, The Netherlands) was connected to the pump (Masterflex, The Netherlands), where 0.89 microbore tubing (Cole Palmer) was used. The four bioreactor systems were placed in a temperature-controlled box and kept at 37 °C, providing per run four scaffolds (*n*=4) with stromal cells for RNA extraction. These incubation units had to be supplied with a gas-controlled atmosphere. To supply the cells with oxygen and carbon dioxide, an oxygenator was built. The oxygenator comprised a closed chamber containing a gas-permeable silicon tube. The gas environment in the chamber was kept at a constant level of 21% O_2_ and 5% CO_2_ and medium was pumped through the gas-permeable tube at a flow rate of 0.3 ml/min. This system maintained the pH (7.1) at the bioreactor outlet during the culture period.

Chemo-optic flow-through micro oxygen sensors (FTC-PSt-3; Presens GmbH, Germany) that detect the quenching of luminescence by oxygen and an oxygen meter (Fibox-3; presens GmbH) were used as previously shown [4]. For 100% dissolved oxygen (DO) calibration, gas with the compositions mentioned above was supplied to the medium via the oxygenator. For 0% DO calibration, nitrogen gas was supplied through the medium via the oxygenator. Flow through cell (FTC-HP8-S, Presens GmbH) connected to pH-1 mini (Presens GmbH) were used to measure the pH of the medium at the outlet of the culture chamber.

### 1.3 Scanning electron microscope **(**SEM**)** photograph

**Fig. 2:**
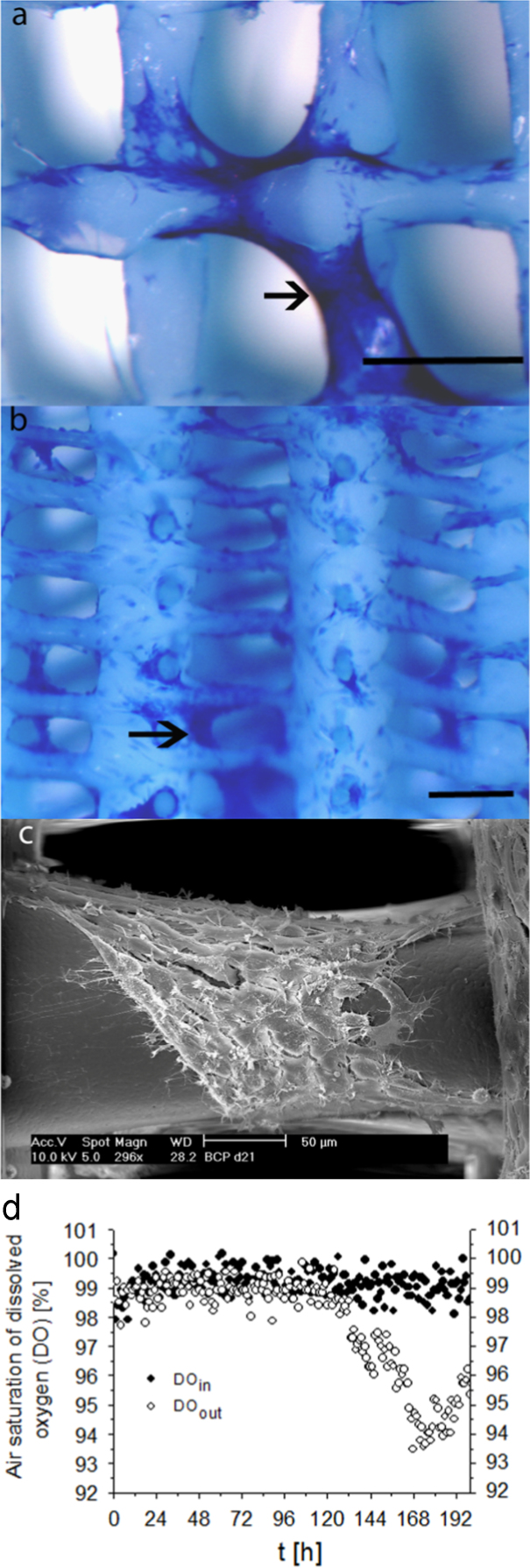
Perfusion bioreactor culture of stem cells in scaffolds. Representative top view (A) and cross section (B) of scaffolds depicting the cell organization of stromal cells (Methylene blue stained) on PEOT/PBT fibers under continuous flow (0.3 ml/min). Scale bar: 500 µm. Arrows on (A and B) point to cells aggregated on fibers as depicted by the scanning electron microscope photograph (C), scale bar: 50 µm. Dissolved oxygen (DO) measurements showing baseline of dissolved O_2_ entering the reactor (DO_*in*_) and dissolved O_2_ exiting the reactor (DO_*out*_), which represents the consumption profile by stromal cells (D). DO data represents the mean (*n*=4 runs).

Scaffolds were collected from the bioreactor system and fixed in 1,5% glutaraldehyde, 0,14 M cacodylic buffer, pH 7,2–7,4 adjusted with 1 M hydrochloric acid (HCl). Scaffolds were then washed with phosphate buffered saline (PBS) and sectioned into smaller 4 pieces. These pieces were dehydrated and dried with a CO_2_ critical point dryer, CPD 030 (Balzers). Scaffold sections were sprayed with gold using a sputter coater, 108Auto (Cressington) before using a scanning electron microscope (SEM), XL 30 SEM FEG (Philips) at 10 kV (Fig. 2).

### 1.4 Glycosaminoglycans **(**GAGs**)** and collagen quantification

GAGs were quantified on 2D discs and 3D scaffolds with 9-dimethylmethylene blue chloride (DMMB, Sigma-Aldrich) staining in PBE buffer: 14.2 g/l Na_2_HPO_4_ and 3.72 g/l Na_2_EDTA, pH 6.5. A micro plate reader (Bio-TEK instruments) was used to spectrophotometrically determine absorbance at 520 nm. Chondroitin sulfate was used as standard.

**Fig. 3:**
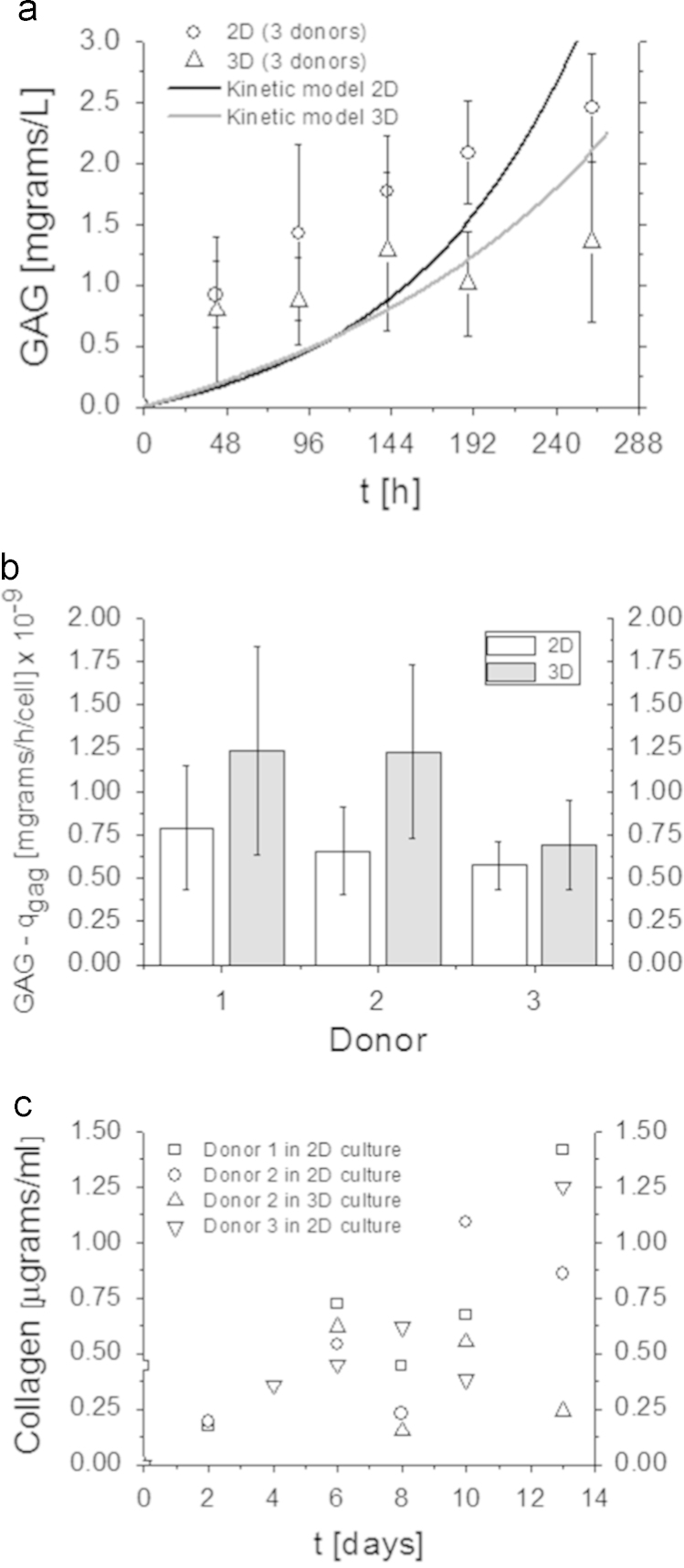
Composition of the extracellular matrix. Glycosaminoglycan-rich matrix is consistently produced in 2D and 3D (A) and at comparable *q*_*GAG*_ rates (B). On the contrary, total collagen was measured in all donors in 2D, but only in one donor in 3D (C). Scale bar: 100 µm. Measurements in time represent the mean and standard deviation (*n*=3 donors, n′=6 samples/donor). *q*_*GAG*_ was estimated with nlinfit and nlparci functions of Matlab with statistical significance set at *p*≤0.05. Standard deviations reflect the fit of the kinetic model when quantifying cell-number-dependent measurements.

Collagen quantification was performed by high-performance liquid chromatography (HPLC). First, samples were hydrolyzed (110 °C, 20–24 h) with 750 µL 6 M HCl. Samples were dried and redissolved in 800 µL of 2.4 mM homoarginine (internal standard for amino acids; Sigma-Aldrich) in water. For amino acid analysis, samples were then diluted 100-fold with 0.1 M sodium borate buffer (pH 8.0). Then, HPLC was performed as described before [5]. Collagen content was calculated from the total amount of hydroxyproline in each sample. It was assumed that there are 300 hydroxyproline residues per collagen molecule with a molecular weight of collagen of 300 kDa.(Fig. 3)

### 1.5 Histological stainings

**Fig. 4:**
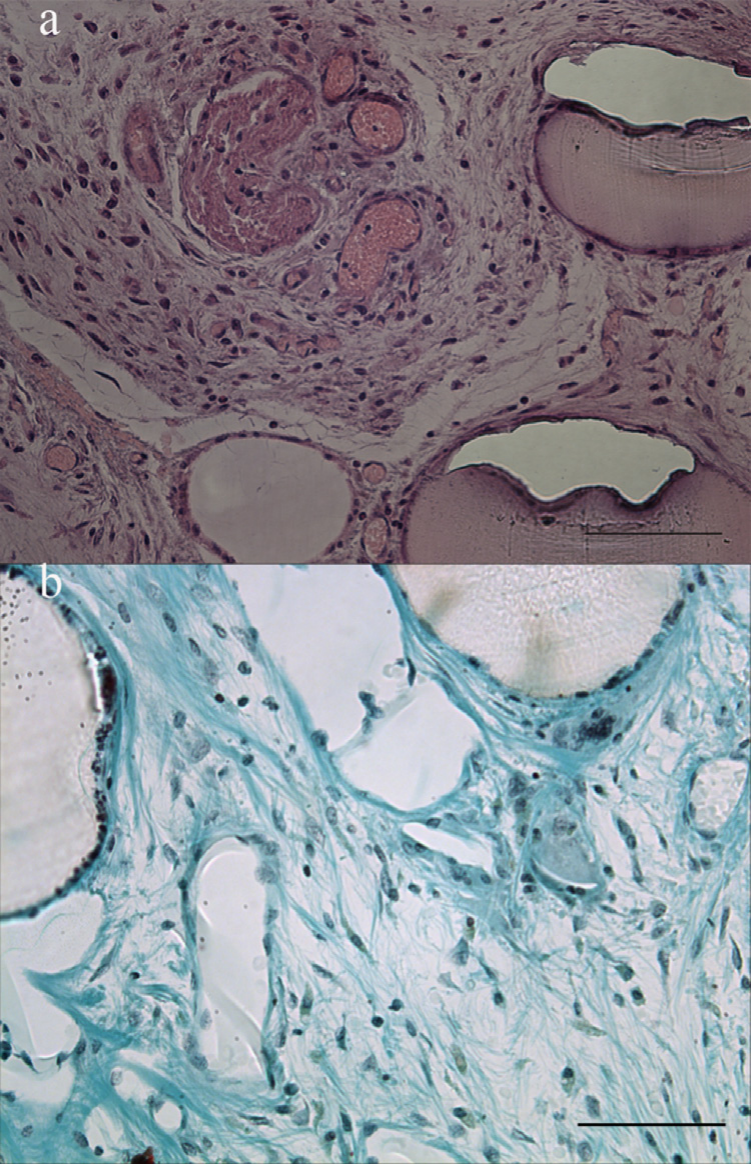
Scaffolds with stromal cells (day 8) were implanted in nude mice for 30 days. The spatiotemporal organization of stromal cells in scaffolds leads to the presence of tissue *in vivo*: Cell (black/brown) and cytoplasm (Red) staining (A, Haematoxylin/Eosin) or keratin (Red), collagen (Blue) and nuclei (brown/black) staining (B, Masson׳s tri-chrome). However, one can note that tissue organization increases with respect to scaffolds (5×10^3^ cells/ml) when cell concentrations are increased to 6×10^6^ cells/ml in implantable wells (Fig. 5). Scale bar: 100 µm.

**Fig. 5:**
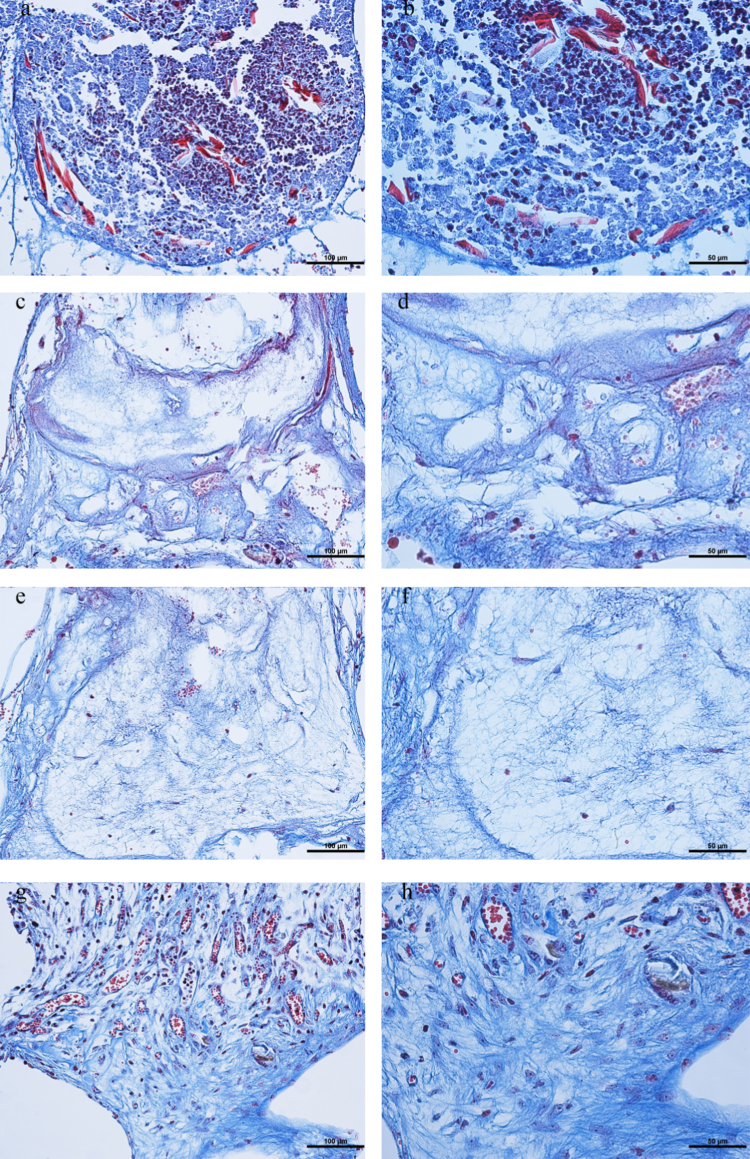
Increasing concentrations of stromal cells *in vivo*. Slides stained with Masson׳s tri-chrome: Keratin (Red), collagen (Blue) and nuclei (dark brown or black) from wells with and without stromal cells implanted in nude mice. The control (A-B, wells without cells) and wells with 3×10^6^ cells/ml (C, D) did not show tissue organization in the wells. Wells with 7×10^6^ cells/ml (E-F) and 14×10^6^ cells/ml (G-H) displayed tissue organization; that is, alignment of collagen fibers (F and H) and blood vessel distribution (E-H). Scale bars: (A, C, E, G=100 µm), (B,D, F, H=50 µm). blue arrow points to collagen fibers. Red arrow points to one blood vessel.

Sections were stained with Masson׳s trichrome (Sigma-Aldrich) to visualize: Collagen, nuclei and cells; and haematoxylin/Eosin (Sigma-Aldrich) to visualize the cytoplasm and nuclei. Mounted slides were examined through light microscopy.(Figs. 4 and 5)

### 1.6 CD31 quantification

(Fig. 6).

### 1.7 STC1 representative histograms

(Fig. 7).

## Figures and Tables

**Fig. 6:**
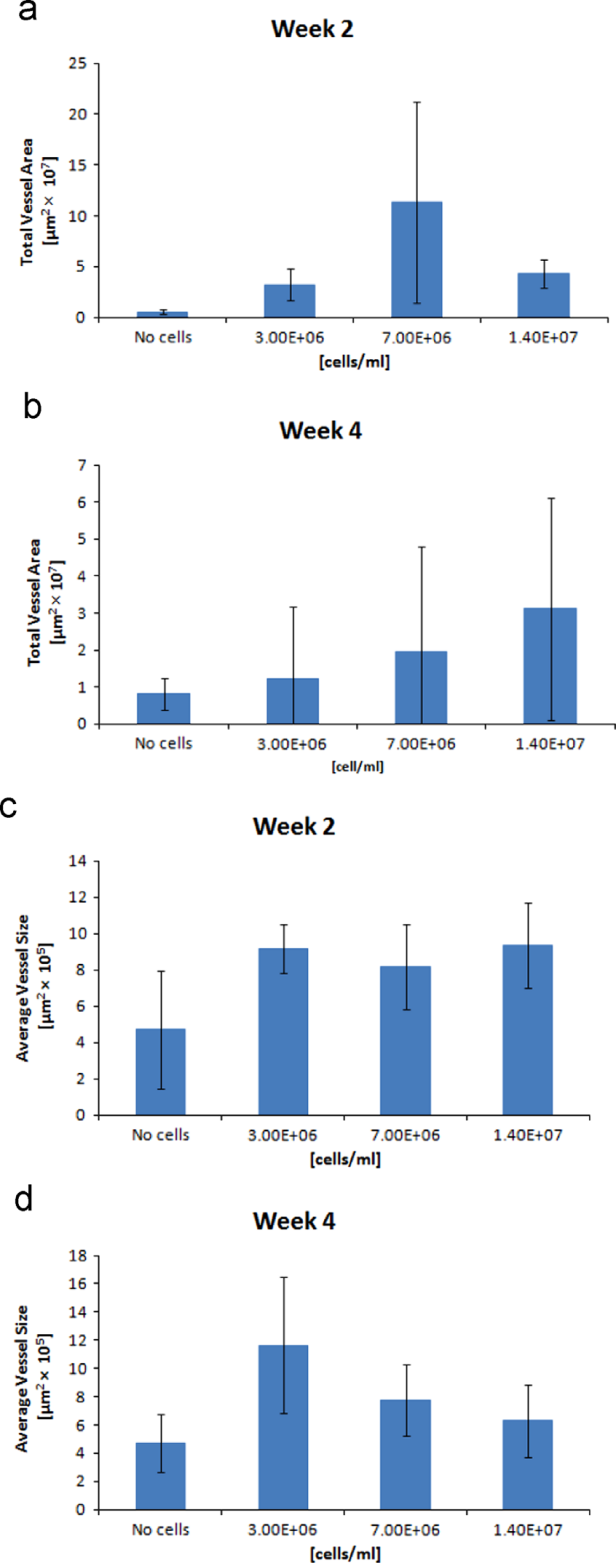
Other parameters quantified through CD31 staining and analyzed with ImageJ. The total vessel area was obtained by adding the area of all individual blood vessels in the section of a well (*n*=7) on week 2 (A) and week 4 (B). On the other hand, the average vessel size represents the mean vessel size found in a well for a particular Stromal cells concentration on week 2 (C) and week 4 (D).

**Fig. 7:**
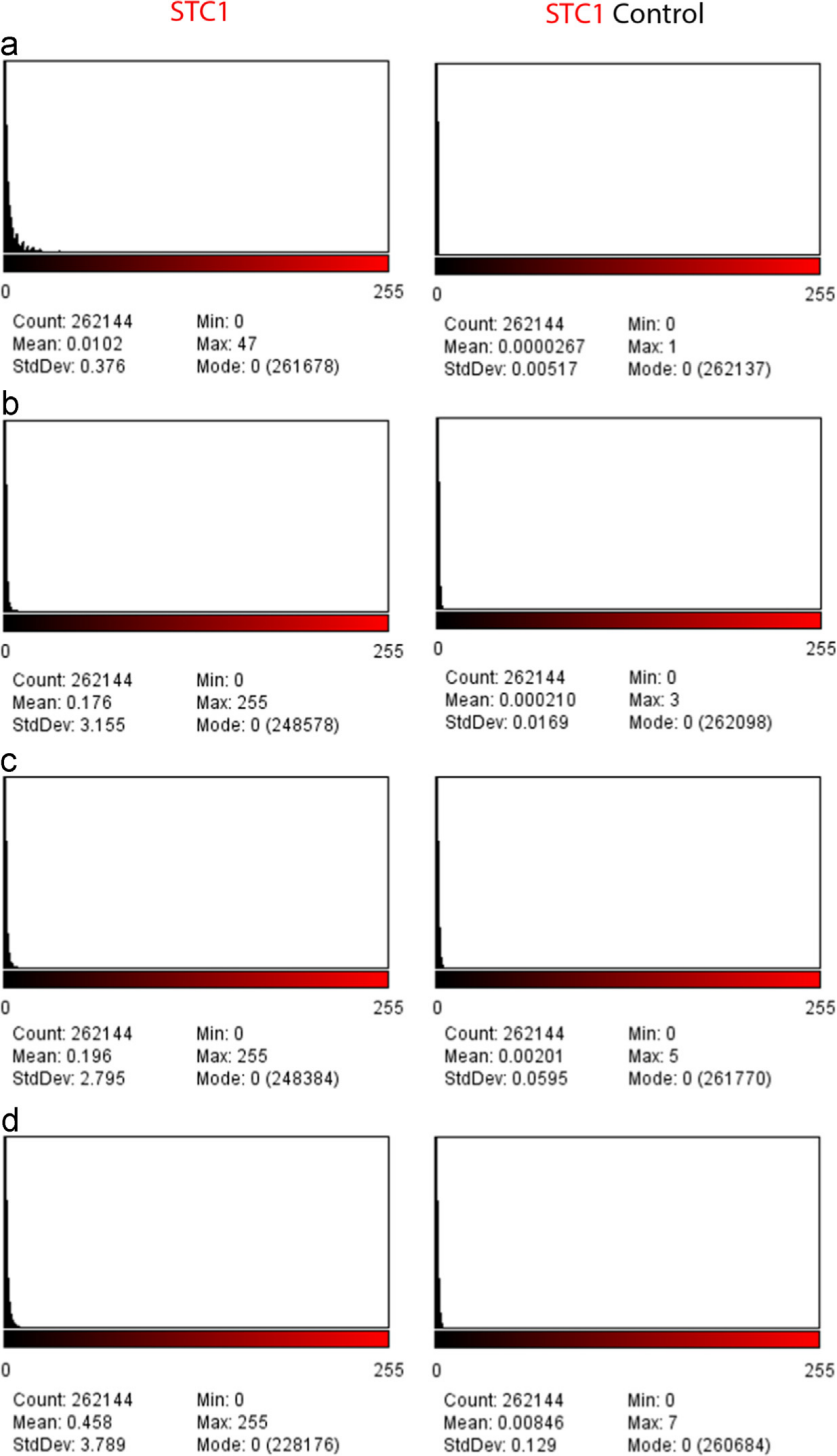
Histograms for well sections treated with the STC1 antibody (Cy5 fluorophore) and control without the STC1 antibody (Cy5 flurophore) are shown. Wells without implanted cells (A), and the wells containing three stromal cell concentrations 3×10^6^ cells/ml (B), 7×10^6^ cells/ml (C), and 14×10^6^ cells/ml (D). The ascending mean intensity can be observed with higher cell concentrations on the left column and the random mean intensity on the right column.

**Table 1:** Seeding efficiencies and seeding densities of three human Stromal cells donors cultured in 2D discs and 3D scaffolds of PEOT/PBT.

	**2D**		**3D**	
**Donor**	**Seeding efficiency [%]**	**Seeding density [cells/cm**^**2**^**] x 10**^**3**^	**Seeding efficiency [%]**	**Seeding density [cells/cm**^**2**^**]×10**^**3**^
**1**	10.93	5.68	15.91	9.01
**2**	12.74	6.62	7.97	4.51
**3**	16.95	8.81	12.84	7.28
**Mean**	13.54	7.04	12.24	6.93
**SD**	3.04	1.60	4.01	2.27

**Table 2:** Constants used in the CFD models.

**Condition\constants**	**Dimensions (mm)**	**Initial glucose concentration (c**_**0**_**, mmol/L)**	**Diffusion coefficient glucose (*****D*****, m**^**2**^**/s)**	**Reaction rate (*****R*****, mol/(m**^**3**^**⁎s))**	**Inward flux (*****N*****, mol/(m**^**2**^**⁎s))**
**Complete 3D scaffold**	φ4×1	4.5	9e−10	1.842e−4	
**3D Pore**	0.65×0.65×1	4.5	9e−10		−1.25e−9
